# Comparison of Acoustic Structure Quantification (ASQ), shearwave elastography and histology in patients with diffuse hepatopathies

**DOI:** 10.1186/s12880-015-0100-1

**Published:** 2015-12-03

**Authors:** Jan Keller, Tanja Eva-Maria Kaltenbach, Mark Martin Haenle, Suemeyra Oeztuerk, Tilmann Graeter, Richard Andrew Mason, Thomas Seufferlein, Wolfgang Kratzer

**Affiliations:** Department of Internal Medicine I, University Hospital Ulm, Albert-Einstein-Allee 23, 89081 Ulm, Germany; Department of Diagnostic and Interventional Radiology, University Hospital Ulm, Albert-Einstein-Allee 23, 89081 Ulm, Germany; Louis Stokes Cleveland Department of Veterans Affairs Medical Center, 10601 East Boulevard, Cleveland, OH 44106 USA; Universitätsklinikum Ulm, Zentrum für Innere Medizin, Klinik für Innere Medizin I, Albert-Einstein-Allee 23, D-89081 Ulm, Germany

**Keywords:** Acoustic Structure Quantification (ASQ), Acoustic Radiation Force Impulse (ARFI), Elastography, Liver fibrosis, Virtual touch

## Abstract

**Background:**

Objective of the study was to evaluate the diagnostic value of novel ultrasonographic modalities in comparison with simultaneously performed liver biopsy.

**Methods:**

The results of simultaneously performed examinations using Acoustic Structure Quantification (ASQ), Virtual Touch Imaging and Quantification (VTIQ) and Virtual Touch Tissue Quantification (VTTQ) were compared with the findings of liver biopsy in patients with a wide variety of diffuse hepatopathies (*n* = 51). The histologically determined fibrosis stage according to Desmet and Scheuer was compared with quantitative measurements returned by the ultrasonographic imaging modalities.

**Results:**

No statistically significant correlation with histologically determined fibrosis stage could be identified for any measurements returned using ASQ. Increasing severity of hepatic steatosis, however, was associated with a reduction in the focal disturbance (FD) ratio (r = −0.55; *p* < 0.0001). The shearwave velocities measured using VTTQ satisfyingly correlated with fibrosis stage (r = 0.56; *p* > 0.0001). Fibrosis stages > F2 were associated with an area under the curve (AUC) of 0.94 (95 %-CI:0.84–0.99). A cut-off value for shearwave velocity of 1.66 m/s was determined with a sensitivity of 100 % and a specificity of 84 %. VTIQ showed a less pronounced but acceptable correlation with fibrosis stage (r = 0.35; *p* = 0.0154). For fibrosis stages > F2 analysis showed an AUC of 0.84 (95 %-CI:0.70–0.93). The cut-off value was 1.82 m/s with a sensitivity of 100 % and a specificity of 58 %.

**Conclusion:**

While ASQ showed no diagnostic advantage in our patient collective, VTTQ showed high reliability for determining severe liver fibrosis in a group of patients with diffuse liver diseases of different etiology.

## Background

The diagnosis and follow-up monitoring of diffuse hepatopathies continues to present modern Medicine with a number of challenges. In many cases, an exact determination of the disease entity at hand requires liver biopsy and remains the gold standard for the diagnosis of diffuse hepatopathies. Histological assessment, however, is crucial not only for confirming the initial diagnosis but remains important for therapeutic management and subsequent monitoring of patients’ progress. For example, the fibrosis stage is one of the most important markers in hepatitis C virus (HCV) infection [1]: the success rate for antiviral therapy depends on the fibrosis stage at time of initiation of therapy [2]. Patients with fibrosis stage 3 disease should in addition be regularly screened for hepatocellular carcinoma (HCC) [3]. The miniscule volume of the liver biopsy cylinder represents only 1/50,000 of the total mass of the liver, which underscores the possibility of misinterpretations in the sense of a sampling error [4]; the reliability of the assessment increases in proportion to the length of the tissue cylinder [5]. Multiple tissue samples or the use of longer puncture needles may help to maximize length, though the latter is associated with an increased risk of bleeding [6]. The need for alternatives brought up a variety of new technologies, like transient elastography, magnetic resonance elastography or later shearwave elastography. Quantifying the stiffness of the liver, would allow to draw conclusions about the extent of liver fibrosis. Both transient elastography and Acoustic Radiation Force Impulse (ARFI) shearwave elastography represent ultrasonographic methods that permit quantitative assessment of liver elasticity. Numerous studies have described a nearly equivalent sensitivity for these two methods in detecting liver fibrosis stages ≤ F2 [7]. Even though transient elastography has been available for a longer period, the need for a devise only dedicated to this examination, restricts clinical practicability. Magnetic response elastography is however expensive in time and limited in its availability. As opposed to this shearwave elastography is integrated in a conventional ultrasonic devise. Another approach to determine liver fibrosis non-invasive was the detection of structural alterations of the liver parenchyma. Acoustic Structure Quantification (ASQ) is a novel modality developed by Toshiba that permits analysis of tissue homogeneity in the liver [8, 9] whose diagnostic value has been discussed in only a few publications to date. Objective of the present study was to evaluate the diagnostic value of ASQ and ARFI in comparison to liver histology in patients with diffuse liver diseases of different etiology.

## Methods

### Study design

A collective of 51 patients with diffuse hepatopathies scheduled to undergo diagnostic liver biopsy were examined by means of elastography and ASQ in the present prospective clinical study. Prior to recruiting patients, the study protocol was approved by the University’s ethics commission according to the Helsinki Declaration. Each patient provided his or her informed written consent to participation in the study. Ultrasonographic examinations were performed on the same day and several hours prior to the planned liver puncture. The examiner was blind to the fibrosis score of the patient, as the pathologist was blind to the ultrasonic measurements. All ultrasonic examinations were performed by a single person. This study was performed without any financial support or other consideration.

### Liver biopsy and histology

Tissue samples were obtained either laparoscopically or under ultrasonographic guidance. Due to accessability, the right hepatic lobe was the source of biopsy materal in most cases. Liver fibrosis was assessed by the hospital’s department of pathology according to Desmet und Scheuer [10] and assigned to one of five stages, ranging from stage F0 (no fibrosis) to stage F4 (liver cirrhosis). Tissue samples used for assessment were at least 10 mm in length and contained at least four portal tracts. Cases diagnosed as intermediate stages were assigned to the next higher stage. In addition, three steatosis stages were defined: grade 0 with lipid inclusions in < 5 % of hepatocytes; grade 1, 5–30 %; and grade 2, >30 %.

### Acoustic Structure Quantification (ASQ)

Acoustic Structure Quantification (ASQ) is a novel feature of the Toshiba Aplio 500 ultrasound system (6C1 convex transducer head) that is intended to facilitate analysis of tissue homogeneity in the liver. This is based on an analysis of the Probability Density Function (PDF) in raw imaging data. Yamaguchi et al. [11, 12] were able to demonstrate experimentally that PDF of echo amplitudes in ideal liver tissue approximates a Rayleigh distribution. A statistical analysis allows the program to detect deviations of the variances of these PDF from the expected Rayleigh distribution. Quantitative analysis generates a chi-square histogram for a defined measurement area. The higher the chi-square value, the greater the deviation from ideal tissue. With the version available to us, macrostructures within the measurement area were detectable with the help of mathematical logarithms. These are presented in a separate curve and can be interpreted as small vascular and connective tissue structures. The relationship between the AUC of these macrostructures and the AUC of the “parenchyma” curve is designated the focal disturbance (FD) ratio. In the present study, a five-second raw data clip of the subcostal longitudinal and cross section was recorded for each patient. Analysis was performed on a separate computer employing the PC-ASQR V1.01R000 software. Here, up to four regions of interest (ROI) were defined in B scan in such a way as to cover the largest area of liver parenchyma possible while avoiding large connective tissue or vascular structures. Measurements from five frames per video clip were compiled. Care was taken that ROIs were placed only in regions with a visually qualified good signal-to-noise ratio.

### Shear wave elastography

The method used in the present study is a subform of shear wave elastography belonging to ARFI elastography. Measurements were acquired using a conventional ultrasound unit (Siemens Acuson S3000), which, together with the Virtual Touch program, allows for quantitative determination of tissue elasticity using either of two different modalities. Virtual Touch Tissue Quantification (VTTQ) allows individual B scan measurements while Virtual Touch Tissue Imaging and Quantification (VTIQ) permits parallel measurements within a primary ROI of freely determined size using a linear transducer. Measurements are color coded depending on shear wave velocity. Six intercostal measurements at 5 cm depth in different areas of the right hepatic lobe were obtained using VTTQ. For VTIQ, the left hepatic lobe was also examined. Here, via an epigastric approach, six measurements 15 mm beneath the liver capsule were acquired while, in the right hepatic lobe, six measurements, each, were obtained at 15 mm and 20 mm beneath the liver capsule.

### Statistical analysis

Statistical evaluation of the data was performed using the “Statistiklabor” (v. 3.81, Free University of Berlin, Germany). All other analyses were performed using the open source “R” software (v. 3.1.2). Following the compilation of data, the medians of the individual measurements were represented for the respective fibrosis stage using a boxplot graph and analyzed for statistically significant differences using the Wilcoxon-Mann–Whitney-Test. Correlation of the individual parameters was tested after calculation of the Spearman rank correlation coefficient “r”. The level of significance was set to α = 0.05. Correlation was differentiated in poor (0 < r < 0.3), acceptable (0.3 ≤ r < 0.5), satisfied (0.5 ≤ r < 0.7), good (0.7 ≤ r < 0.9) and excellent (r ≥ 0.9). In addition, the Receiver Operating Characteristics (ROC) curves were determined using the Acomed “ROC Curve” and the Area Under the Curve (AUC) was calculated.

## Results

Tables 1 and 2 presents an overview of the patient collective and other measurement parameters. Because of the small number of patients diagnosed as being in fibrosis stage F4, these patients were grouped together with patients with stage F3 disease. Four of 51 patients were excluded from the analysis due to missing or incomplete biopsy findings.

**Table 1:** Overview of the subject collective and individual measurement parameters

	N (%); mean ± STD
Number of subjects	51
Gender	
Male	31 (61 %)
Female	20 (39 %)
Age (years)	51 ± 11
Weight (kg)	75 ± 14
Height (m)	1.7 ± 0.1
BMI (kg/m^2^)	25.5 ± 3.5
Hepatopathies	
Hepatitis C	13 (25 %)
Hepatitis B	5 (10 %)
Non-alcoholic Steatohepatitis (NASH)	10 (20 %)
Non-alcoholic fatty liver disease (NAFLD)	6 (12 %)
Autoimmune Hepatitis (AIH)	5 (10 %)
Toxic liver damage	3 (6 %)
Other and Unknown	9 (17 %)
Fibrosis stage (Desmet/Scheuer)	
Stage F0	15 (32 %)
Stage F1	10 (21 %)
Stage F2	13 (28 %)
Stage F3 and F4	9 (19 %)
Tissue cylinder	
Length (cm)	1.8 ± 0.6
Portal tracts	7 ± 3
Hepatocytes with lipid inclusions	
Grade 0: <5 %	34 (67 %)
Grade 1: 5–30 %	10 (20 %)
Grade 2: >30 %	7 (13 %)

**Table 2:** Overview of the measured parameters with VTTQ (Virtual Touch Tissue Quantification), VTIQ (Virtual Touch Tissue Imaging & Quantification) and ASQ (Acoustic Structure Quantification). Wilcoxon-Mann–Whitney-test was used to differentiate the groups. The level of significance was set to α = 0.05

	Fibrosis stage	Mean ± STD	Wilcoxon-Mann–Whitney-test against fibrosis stage		
			F1	F2	F3 and F4
VTTQ righ liver lobe, 5 cm deep		Shearwave velocity [m/s]			
	F0	1.37 ± 0.37	*p =* 0.698	*p =* 0.153	*p* < 0.001
	F1	1.37 ± 0.26	---	*p =* 0.153	*p* < 0.001
	F2	1.62 ± 0.81	*p =* 0.153	---	*p =* 0.002
	F3 and F4	2.61 ± 0.73	*p* < 0.001	*p =* 0.002	---
VTIQ right liver lobe, 1.5 cm under liver capsule		Shearwave velocity [m/s]			
	F0	1.91 ± 0.44	*p =* 0.254	*p =* 0.482	*p =* 0.022
	F1	1.64 ± 0.27	---	*p =* 0.710	*p =* 0.003
	F2	1.97 ± 0.39	*p =* 0.482	---	*p =* 0.002
	F3 and F4	2.42 ± 0.58	*p =* 0.003	*p =* 0.002	---
ASQ right liver lobe, longitudinal section		Focal Disturbance - Ratio			
	F0	0.18 ± 0.13	*p =* 0.764	*p =* 0.505	*p =* 0.717
	F1	0.19 ± 0.16	---	*p =* 0.443	*p =* 0.386
	F2	0.17 ± 0.17	*p =* 0.443	---	*p =* 0.800
	F3 and F4	0.17 ± 0.19	*p =* 0.386	*p =* 0.800	---

### Acoustic Structure Quantification (ASQ)

**Fig. 1:**
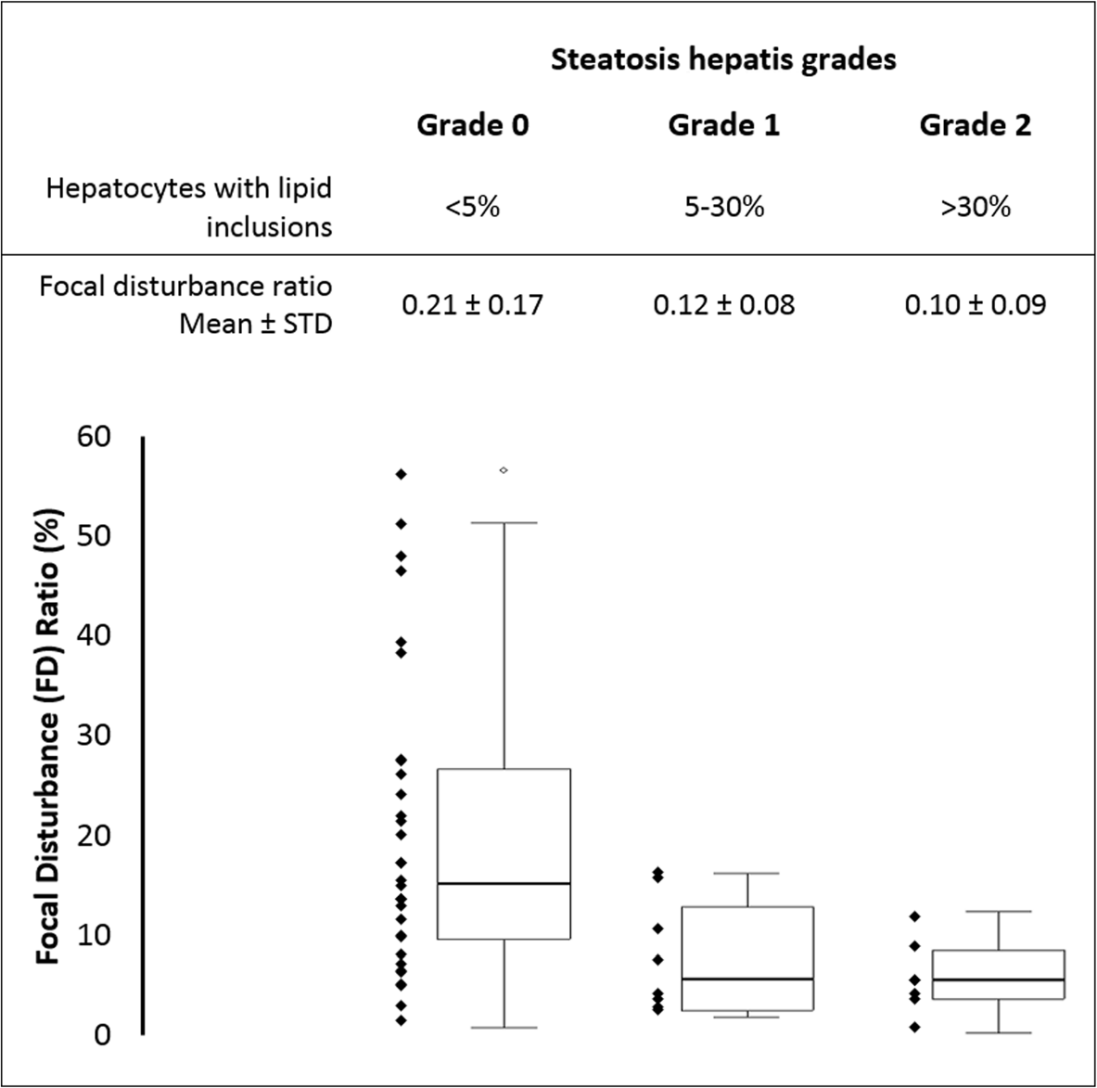
Focal Disturbance (FD) Ratio of liver cross sections grouped according to the respective steatosis grade measured using Acoustic Structure Quantification (ASQ). Grade 0: < 5 % of hepatocytes with lipid inclusions, Grade 2: 5–30 %, Grade 3: >30 %

All measurements were successfully performed. For the correlation of the longitudinal and cross sections of the liver, a Spearman correlation coefficient of r = 0.61 was returned for the modes of the chi-square histogram (*p* < 0.001); r = 0.73 for the medians (*p* < 0.0001) and r = 0.75 for the FD ratios (*p* < 0.0001). None of these measurement parameters were found to correlate in any way with the fibrosis stage, nor did differentiation of the fibrosis stage based on the measurement parameters using the Wilcoxon-Mann–Whitney Test yield statistically significant results. However, for both the longitudinal and cross sections, our findings did show a negative, satisfied correlation with the degree of hepatic steatosis for the modes (r = −0.50; *p =* 0.0003), means (r = −0.53; *p =* 0.0001) and the FD ratios (r = −0.60; *p* < 0.0001). Considering only those subjects without hepatic steatosis, our findings similarly fail to support a statistically significant correlation of ASQ measurements with fibrosis stage. Figure 1 shows the FD-ratios of the liver cross sections in relation to the corresponding degree of steatosis.

### Shearwave elastography

**Fig. 2:**
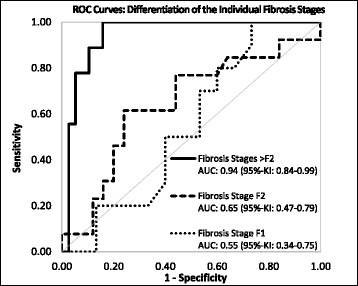
Receiver Operating Characteristics (ROC) curve for differentiation of the individual fibrosis stages according to Desmet and Scheuer based on the corresponding shear wave velocities. *AUC: Area Under the Curve; 95 %-CI: 95 % Confidence Interval*

The results of the VTTQ measurements show a satisfied correlation with r = 0.56 (*p* < 0.0001) with respect to the stage of fibrosis. For VTIQ measurements in the right hepatic lobe, an r values of 0.35 (*p =* 0.0154) and a poor correlation with r = 0.25 (*p =* 0.1094) were achieved for measurements at 15 mm and 20 mm beneath the capsule, respectively, while, for the left hepatic lobe, an r = 0.16 (*p =* 0.2913) was calculated for measurements at a depth of 15 mm. A statistically significant difference in VTTQ measurements was demonstrated using the Wilcoxon-Mann–Whitney test between patients with fibrosis stages F3 and F4 compared with all other stages (*p* < 0.003). Similar findings were returned for VTIQ measurement of the right hepatic lobe at 20 mm depth (*p* < 0.023) but not in the left hepatic lobe at 15 mm depth and only for stages F0 and F1 in the right hepatic lobe at a depth of 15 mm. No statistically significant differences could be shown for stages F0 to F2. The AUC values and cut-off values determined from the ROC curves are shown in Table 3. Figure 2 presents the corresponding ROC curves for differentiation of the respective fibrosis stages.

**Table 3:** “Area Under the Curve” (AUC) values calculated from a Reciever Operating Characteristics (ROC) curve for differentiation of the individual fibrosis stages according to Desmet and Scheuer based on measured shear wave velocities

		Fibrosis stage		
		Stage F1	Stage F2	Stage > F2
VTTQ	AUC (95 %-CI)	0.55 (0.34–0.75)	0.65 (0.47–0.79)	0.94 (0.84–0.99)
	Cut Off (Sensitivity/Specificity)	1.21 m/s (80 %/40 %)	1.36 m/s (77 %/56 %)	1.66 m/s (100 %/84 %)
VTIQ 15 mm depth Left hepatic lobe	AUC (95 %-CI)	-	-	0.82 (0.67–0.92)
	Cut Off (Sensitivity/Specificity)	-	-	1.90 m/s (83 %/65 %)
VTIQ 15 mm depth Right hepatic lobe	AUC (95 %-CI)	-	-	0.84 (0.70–0.93)
	Cut Off (Sensitivity/Specificity)	-	-	1.82 m/s (100 %/58 %)
VTIQ 20 mm depth Right hepatic lobe	AUC (95 %-CI)	-	-	0.87 (0.73–0.95)
	Cut Off (Sensitivity/Specificity)	-	-	1.60 m/s (100 %/62 %)

*VTTQ * Virtual Touch Tissue Quantification, *VTIQ* Virtual Touch Tissue Imaging & Quantification, *CI* Confidence Interval

In addition, other factors were assessed for their influence on shearwave velocities. Ignoring the fibrosis stage, it was possible to detect an acceptable statistically significant correlation for bile duct diameter (r = 0.33; *p =* 0.0195) and for the degree of inflammation according to Desmet/Scheuer (r = 0.42; *p =* 0.0034). Limiting the collective to the lower fibrosis stages (< F2), these correlations were no longer observed and we also found no significant correlation for other parameters, such as portal vein diameter or mean portal vein flow. The diameter of the common bile duct (CBD; r = 0.33; *p =* 0.0238) and the degree of inflammation (r = 0.78; *p* < 0.0001) also correlated acceptably, respectively well with the fibrosis stage and thus possibly only apparently with the shearwave velocities. No significant correlation of the measurements with other parameters could be demonstrated.

## Discussion

### Acoustic Structure Quantification (ASQ)

To date, only a few studies investigating the relatively new Acoustic Structure Quantification (ASQ) technique have been published. The influence of hepatic steatosis on measurements observed in the present study had already been reported by Kuroda et al. in leptin-deficient mice [13]. Those authors postulated that the increasing number and size of fat droplets overlying microscopic interferences leads to a homogenization of the ultrasound image. Because this correlation was identified in our data for the medium and high values in the chi-square histogram, a co-existing hepatic steatosis might lead to interferences affecting fibrosis detection. Ricci et al. [8] and Toyoda et al. [9] were able to demonstrate statistically significant differences for the chi-squared values of the individual fibrosis stages. Ricci et al. [8] mention the influence on measurement results caused by hepatic steatosis but the prevalence within their subject collective remains unclear. In the study of Toyoda et al. [9], no cases of severe hepatic steatosis (>30 % of hepatocytes with lipid inclusions) were observed. If, in the present study, those patients with hepatic steatosis had been excluded from the analysis, there would also have been no statistically significant differences. A current publication by Krämer et al. [14] similarly found no statistically significant correlation of ASQ measurement parameters with fibrosis stage. Here, again, precise data regarding the prevalence of hepatic steatosis in the study collective were not reported. In addition, both Krämer et al. and the other two studies cited above utilized a version of the ASQ software that differed from that used in the present study: whereas Toyoda et al. [9] used a prototype they had themselves developed, both Ricci et al. [8] and Krämer et al. [14] used commercially available, though older, versions of the ASQ software. It is impossible to estimate to what extent the results of the respective studies depend on the respective software versions. Furthermore, inaccuracies in data collection cannot be excluded. There are no established uniform measuring points concerning ASQ analysis until now. Our approach was to try to cover as much liver parenchyma with the ROIs as possible, while avoiding sizeable vessels. Up to four ROIs can be placed, but because they are of a fixed form and stretching out in relation to the fanning out of the ultrasonic picture, accurate placement of the ROI is aggravated. This led to the use of smaller ROIs, thus covering a smaller amount of the liver parenchyma. Including a small vessel can lead to significant differences of reran measurements. Since according to Ricci et al. [8] results the differences in Cm^2^-values of the fibrosis stages are small in comparison to the range of measured values, the slightest variations can degenerate the diagnostic value. The algorithm discriminating Rayleigh- and non-Rayleigh-elements, additionally causes inaccuracies depending on the cut-off value and therefore compounds data interpretation. Assessment errors at the histopathological examination are also possible. With only 51 patients, the subject collective in the present study is relatively small and no data from a healthy subject collective were available for comparison. The results of the present study fail to support a diagnostic advantage for ASQ.

### Virtual Touch Tissue Imaging & quantification (VTIQ)

Virtual Touch Imaging & Quantification (VTIQ) was originally designed for the study of superficial structures and has not yet been widely studied as a method for assessing the liver. Leschied et al. [15] investigated the method for evaluating hepatopathies in infants and found statistically significant increases in shear wave velocities in cases of congenital bile duct atresia compared with other hepatopathies. The limitations that were observed in the present study related primarily to the reduced penetration depth of the high-frequency linear transducer head. Although the technical penetration depth is supposed to be up to 6 cm, depending on the attenuation of the ultrasound by the subcutaneous fat tissue we partially experienced difficulties even measuring at a total depth of 4 cm. While our findings did show VTIQ to be capable of differentiating higher fibroses stages, the greater duration of the measurement, as compared with VTTQ, makes the modality more susceptible to measurement artifacts. The limitations of measurement depth may, depending on the patient’s physical habitus, restrict measurements to the immediate subcapsular zone, which may similarly falsify the measurements. VTIQ, therefore, may be of use in assessing the elasticity of superficial structures and in pediatric cases. With respect to the diagnosis of diffuse hepatopathies, VTTQ appears to be the better choice especially in adults.

### Virtual Touch Tissue Quantification (VTTQ)

Shear wave elastography has been extensively researched. Hence, there is already a large literature consisting of published studies and several larger, pooled meta-analyses [16, 17] regarding ARFI technology’s role in detecting fibrosis. Nierhoff et al. [17] have confirmed its high reliability (AUC = 0.89) for differentiation of fibrosis stages > F2. In their study, the cut-off value for severe fibrosis stood at 1.61 m/s, which differs only negligibly from the cut-off value of 1.66 m/s observed in the present study. An accurate differentiation of fibrosis stage F1 proved impossible in any of the included studies. Reasons for the discrepancy of some of the findings certainly include the quality requirements established for the liver biopsy specimens in the respective studies. The meta-analysis of Bota et al. [16] showed that, while some studies did not define a minimum length for the tissue cylinder or a minimum number of portal tracts, others required a minimum length of 2 cm with at least 10 evaluable portal tracts. This latter quality standard could not be maintained for the present study and had to be loosened in order to obtain a larger number of cases, however increasing the risk of erroneous interpretation by the pathologist. Many studies established a maximum quotient of the interquartile ratio (IQR) to the median of 30 % to minimize measurement errors [3]. In contrast to other studies measuring repeatedly at the same location, we decided to use different measurement locations at comparable depth and went without the 30 % limit. In this way we used the possibility of a widespread examination of the liver to our advantage and overcome the local limitations of a single liver biopsy.

For example, in one subject, an IQR/median quotient of 104 % was calculated. Histopathology findings only showed minimal fibrosis, while the laparoscopy report described scattered fibrotic scars as a sign of incipient fibrosis. Three of a total of six measurement values lay below 1.57 m/s, while the remaining three were in excess of 2.42 m/s. This gap would suggest that the high values were due to the focal tissue scars. A reproducible measurement variation could thus provide diagnostically valuable data and can help to evaluate the histological findings. This has also been proven by another case, in which a patient with shearwave velocities above 4 m/s was histologically diagnosed only with moderate fibrosis. However the laparoscopical findings of this patient stated an extensive alteration of liver parenchyma compatible with liver cirrhosis. This and the possibility of a follow-up examination emphasize how elastographic examination can achieve a diagnostic benefit, in particular if it is combined with the histological findings.

## Conclusions

Based on this examination and other studies' findings the diagnostic value of ASQ still remains doubtful, because of the mentioned difficulties in data acquisition and the proven influence of steatosis hepatis. On the other hand even if shear wave elastography isn't able to replace primary liver biopsy, combined with it, it can enhance clincal validity. Further on its use as an individual follow-up examination might help to reduce the amount of necessary restaging biopsies in patients with chronic liver disease. Regarding VTIQ, it might be better suited for superficial organs like thyroid gland or lymph nodes. However its use in pediatric examination should be further investigated.

## Abbreviation

ASQ: acoustic structure quantification
VTIQ: virtual touch tissue imaging quantification
VTTQ: virtual touch tissue quantification
AUC: area under the curve
CI: confidence interval
HCV: Hepatitis C virus
HCC: hepatocellular carcinoma
ARFI: acoustic radiation force impulse
FD: focal disturbance
ROI: region of interest
ROC: receiver operating curve
IQR: interquartile range
